# Testicular Plasmacytoma as the First Manifestation of Systemic Multiple Myeloma

**DOI:** 10.3390/diagnostics16071101

**Published:** 2026-04-06

**Authors:** Patricia Rodriguez-Parras, Alberto Zambudio-Munuera, Miguel Herraez-Marcos, Francisco Gutierrez-Tejero, Miguel Angel Arrabal-Polo

**Affiliations:** 1 Urology Department, San Cecilio University Hospital, 18016 Granada, Spain; 2 IBS Granada-Instituto Investigacion Biosanitaria de Granada, 18016 Granada, Spain

**Keywords:** testicular mass, testicular plasmacytoma, extramedullary disease, plasma cell neoplasm, ultrasound, PET-CT, hematology

## Abstract

Multiple myeloma is a hematological malignancy characterized by clonal proliferation of plasma cells, usually confined to the bone marrow. Extramedullary disease (EMD) occurs in 7–18% of patients during the disease course and is associated with poor prognosis. Among extramedullary sites, testicular involvement is extremely rare, with an incidence of less than 2%. We present a rare case of testicular plasmacytoma as the first manifestation of systemic multiple myeloma, highlighting its imaging features and clinical implications.

**Figure 1:**
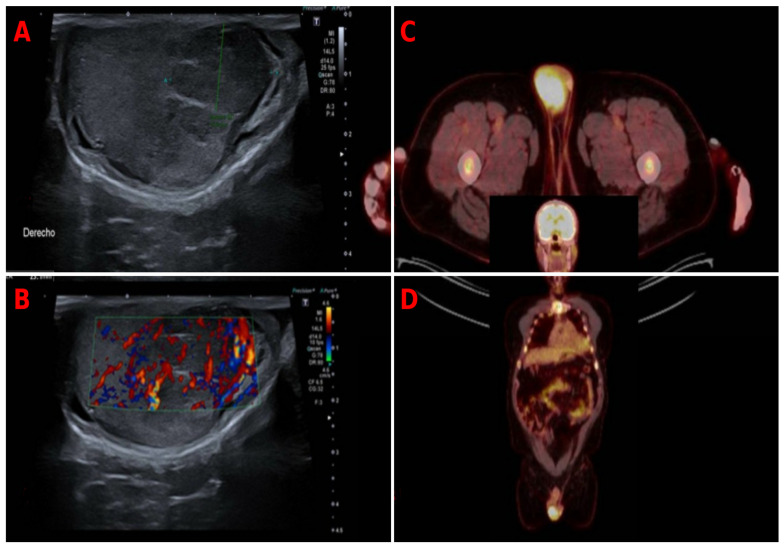
Imaging findings in a patient with testicular plasmacytoma as the first manifestation of multiple myeloma. Multiple myeloma is a hematological malignancy characterized by the clonal proliferation of plasma cells, usually confined to the bone marrow. Extramedullary disease (EMD) occurs in 7–18% of patients during the disease course and represents a poor prognostic marker. Among extramedullary sites, testicular involvement is extremely rare, with an incidence <2% [1,2,3]. (**A**) Gray-scale scrotal ultrasound showing an enlarged right testis with multiple hypoechoic nodules. (**B**) Color Doppler ultrasound demonstrating marked intralesional vascularity. (**C**) Axial PET-CT image showing a hypermetabolic right testicular lesion. (**D**) Whole-body PET-CT revealing multiple hypermetabolic skeletal lesions without lymph node or visceral involvement. A 65-year-old male, ex-smoker with a history of hypertension, presented with a progressively enlarging right testicular mass over a 3-month period. The lesion was painless and not associated with systemic symptoms. Scrotal ultrasound revealed an enlarged right testis with multiple hypoechoic nodules up to 23 mm, showing marked internal vascularity (**A**,**B**). The left testis was atrophic with a mild varicocele. PET-CT demonstrated a hypermetabolic right testicular mass (SUVmax 10.2) and multiple skeletal lesions, without evidence of lymph node or visceral involvement (**C**,**D**). Laboratory studies showed abnormal serum protein electrophoresis with a monoclonal spike, confirmed by immunofixation as IgA lambda. A serum-free light chain assay revealed an abnormal lambda predominance. Beta-2 microglobulin and LDH levels were elevated, while albumin levels were reduced. Renal function showed mild impairment (elevated creatinine). Bone marrow biopsy demonstrated 60% infiltration by clonal IgA lambda plasma cells. According to the Revised International Staging System (R-ISS), the patient was classified as stage II/III (depending on LDH/cytogenetics if available). Right radical orchiectomy was performed. Histopathological examination revealed diffuse infiltration by atypical plasma cells, positive for CD138 with lambda light chain restriction, confirming testicular plasmacytoma. Although cytogenetic data were not available in this case, extramedullary disease in multiple myeloma is frequently associated with high-risk cytogenetic abnormalities such as del(17p), t(4;14), t(14;16), and 1q gain/amplification. These alterations are linked to aggressive disease behavior and poorer outcomes [2]. Extramedullary involvement is thought to result from loss of bone marrow dependence, clonal evolution with acquisition of aggressive features, and the ability of malignant plasma cells to survive in immune-privileged sites such as the testis. Testicular involvement in multiple myeloma is associated with an aggressive clinical course, poor response to therapy, and reduced overall survival [3,4,5,6].

## Data Availability

The data presented in this study are available on request from the corresponding author. The data are not publicly available due to privacy or ethical restrictions due to our legislation.

## Abbreviations

The following abbreviations are used in this manuscript:

EMD	Extramedullary Disease
PET-CT	Positron Emission Tomography–Computed Tomography
SUVmax	Maximum Standardized Uptake Value
IgA	Immunoglobulin A
LDH	Lactate Dehydrogenase
R-ISS	Revised International Staging System
